# Designing qualitative research with value in the clinical and epidemiological context: what, why and how

**DOI:** 10.1093/ckj/sfae422

**Published:** 2024-12-19

**Authors:** Yvette Meuleman, Eline Schade van Westrum, Willem Jan W Bos, Simon P Mooijaart, Marjolijn van Buren, Giovanni Tripepi, Vianda S Stel, Kitty J Jager, Carmine Zoccali, Friedo W Dekker

**Affiliations:** Department of Clinical Epidemiology, Leiden University Medical Center, Leiden, The Netherlands; Department of Clinical Epidemiology, Leiden University Medical Center, Leiden, The Netherlands; Department of Internal Medicine (Nephrology), Leiden University Medical Center, Leiden, The Netherlands; Department of Internal Medicine, St Antonius Hospital, Nieuwegein, The Netherlands; Department of Internal Medicine, section of Gerontology and Geriatrics, Leiden University Medical Center, Leiden, The Netherlands; Department of Internal Medicine (Nephrology), Leiden University Medical Center, Leiden, The Netherlands; Department of Internal Medicine, Haga Hospital, The Hague, The Netherlands; CNR-IFC, Clinical Epidemiology of Renal Diseases and Hypertension, Reggio Calabria, Italy; ERA Registry, Department of Medical Informatics, Amsterdam UMC location University of Amsterdam, Amsterdam, The Netherlands; Amsterdam Public Health Research Institute, Quality of Care, Amsterdam, The Netherlands; ERA Registry, Department of Medical Informatics, Amsterdam UMC location University of Amsterdam, Amsterdam, The Netherlands; Amsterdam Public Health Research Institute, Quality of Care, Amsterdam, The Netherlands; Renal Research Institute, New York, NY, USA; Institute of Biology and Molecular Medicine (BIOGEM), Ariano Irpino, Italy; Department of Clinical Epidemiology, Leiden University Medical Center, Leiden, The Netherlands

**Keywords:** clinical research, epidemiological research, kidney disease, patient-centered research, qualitative research

## Abstract

Clinical and epidemiological research is indispensable for improvements in evidence-based healthcare and health outcomes, but it also leaves important gaps in our understanding of health and illness. Qualitative research has been increasingly recognized as a key to addressing some of these gaps, using both exploratory (to gain a more complete and in-depth understanding of problems) and explanatory (to explain quantitative results) approaches. By finding out ‘what's going on’ and bringing people's stories to light, qualitative research is widely advocated as crucial in enhancing patient-centered research and healthcare. To date, most clinicians, clinical researchers and epidemiologists are relatively unfamiliar with and untrained in qualitative research—a type of research that, compared with quantitative research, requires different research skills and uses a different jargon, type of reasoning, and methods. This article aims to equip them with the basic knowledge necessary to appraise and design qualitative research. Specifically, we provide a comprehensive overview of (i) what qualitative research is, including various examples of qualitative research questions and explanations of the contrasting properties of quantitative and qualitative research; (ii) what constitutes the added value of qualitative research in the clinical and epidemiological context, illustrated using numerous research studies conducted within nephrology; and (iii) practical guidelines for designing qualitative research within this context, including a self-developed checklist containing essential information to include in qualitative research protocols. In doing so, we hope to enrich clinical and epidemiological research with complementary qualitative evidence—amongst others, invaluable insights into patients’ lived experiences and perceptions—and thereby greatly enhance patient-centered research and evidence-based healthcare.

## INTRODUCTION

Clinical and epidemiological research encompasses a wide range of human health-related research, amongst others, focusing on effects of treatments on health outcomes, and focusing on occurrences, patterns, causes, predictors, and effects of health, disease and illness. Results are used to guide strategies to prevent illness and to improve care for and outcomes of patients with diseases [1]. Traditionally, clinical and epidemiological research uses quantitative research methods, such as clinical trials and cohort studies, to answer research questions (e.g. *whether* a treatment or *how much* a risk factor impacts health outcomes) [2, 3].

Notwithstanding the great power of quantitative methods to improve evidence-based healthcare and health outcomes, they still leave important gaps in our understanding of health and illness. For instance, answers to exploratory and explanatory questions such as *what* makes people change their behavior, *how* are people affected by illness, and *why* are some people not satisfied with their healthcare? By finding out ‘what's going on’ and bringing peoples’ stories to light, qualitative research provides previously unknown and in-depth knowledge about, amongst others, people's experiences, perspectives, feelings, needs, preferences and motivations—all of which can drive and explain people's responses to illness, behaviors, decision-making, treatment effects and health outcomes [4–9]. By exploring health and illness as perceived by people themselves and by exploring healthcare as experienced by all stakeholders (including patients and healthcare professionals such as clinicians), it can greatly enhance patient-centered research and contribute to evidence-based healthcare (e.g. provide systematic and high-quality substance to the pillar ‘patient values’ and the pillar ‘clinical expertise’ [4, 5, 10–12].

The value of qualitative research for healthcare research has been increasingly recognized over the past decades. Increases in interest in and publications of qualitative research are seen across many different medical fields, including nephrology [6–9, 12, 13]. However, many clinicians, clinical researchers and epidemiologists are relatively unfamiliar with and untrained in qualitative research [4, 6, 14], a research type that requires different research skills and uses a different jargon, type of reasoning and methods. This article aims to equip clinicians, clinical researchers and epidemiologists with the basic knowledge necessary to appraise and design qualitative research. Specifically, we provide a comprehensive overview of qualitative research, its value in the clinical and epidemiological context, and practical guidelines for designing qualitative research within this context, illustrated using research conducted within nephrology.

## WHAT: WHAT IS QUALITATIVE RESEARCH?

Qualitative research, rooted in social sciences like psychology, has been evolving since the 19th century [15]. In its nature, qualitative research ‘involves the collection and analysis of non-numerical (descriptive) data, to understand a complex phenomenon within the context that it occurs’ [16]. It often entails constructing a rich description and/or explanatory (conceptual) framework or theory in order to deepen our understanding of a topic, and it is especially useful to gain an in-depth understanding of perceptions, behaviors and/or experiences (and the meaning attached to them) [15, 17–19]. Who is being studied can range from a single individual or small group to multiple organizations or a large institution [20].

Different types of research answer different research questions. Quantitative research is mostly based on deductive reasoning (i.e. theory/hypothesis-testing): it aims to test well-specified hypotheses—describing hypothesized relationships, based on existing literature, between predetermined independent and dependent variables—by employing statistical modeling in sufficiently large (random) populations selected using probability sampling. This is suitable to answer ‘whether’ questions (e.g. *whether* a low-sodium self-management intervention improves health outcomes of patients with chronic kidney disease (CKD) [21]), and ‘how much’ questions and variations on this question (e.g. *how much* do symptom burden and quality of life change after dialysis initiation? [22, 23]; *how strong* are depressive symptoms associated with adverse health outcomes in CKD? [24]; and *how often* does hospital readmission occur after kidney transplantation? [25]). To answer such questions, researchers need to quantify data and adopt a reductionistic, simplified perspective on the topic(s): occurrences are counted (e.g. to calculate prevalence estimates) and variables are measured using standardized processes and instruments (e.g. extract data from electronic health records and collect answers to close-ended survey questions) [6, 15, 20, 26].

Contrarily, qualitative research is predominantly based on inductive reasoning (i.e. theory-building/hypothesis-generating): it aims to develop rich descriptions, novel conceptual frameworks and/or theories derived directly from the data [4, 5, 26, 27]. It focuses on finding out ‘what's going on’ and is suited to answer exploratory and explanatory ‘what’, ‘why’ and ‘how’ questions (e.g. *what* are patients’ and healthcare professionals’ experiences with and perspectives on discussing patient-reported outcome measures (PROMs) results in dialysis care? [28]; *why* do some older CKD patients (not) succeed in maintaining self-management regimens? [29]; and *how* do patients experience undergoing dialysis treatment? [30]; for more example qualitative research questions, see Table 1) [15, 31]. To answer such questions, researchers need to adopt a holistic, open perspective on the topic(s); collect data sufficiently rich in details and context (e.g. use open-ended discussions) in order to fully capture human complexities in their wider social, cultural and political context; and maximize the phenomena's complete representation (e.g. select purposively heterogeneous populations using non-probability sampling; see also **How**) [6, 15, 20, 31].

**Table 1:** Example qualitative research questions within the clinical and epidemiological nephrology context, with corresponding basic methods elements.

Research questions starting with ‘What’, ‘How’ and ‘Why’^a^	Basic methods elements
**What** are patients’ and healthcare professionals’ experiences with and perspectives on discussing PROMs results as part of routine dialysis care? [28]	Individual semi-structured interviews with patients and healthcare professionals; purposive sampling; inductive approach using thematic analysis.
**What** illness perceptions underlie patients’ personal experiences and ability to cope with chronic kidney disease, and **how** do these illness perceptions evolve over time? [32]	Individual semi-structured interviews with patients and healthcare professionals; purposive sampling; hybrid inductive approach (using thematic analysis) and deductive approach (using existing self-regulation theory).
**What** are prioritized outcomes and research topics for patients with chronic kidney disease and their caregivers, and **why** are they considered important? [51, 93]	Focus groups with patients and caregivers; purposive sampling; inductive approach using thematic analysis.
**What** are patients’ and healthcare professionals’ experiences with and perspectives on benefits, facilitators and barriers for a nephrology-tailored geriatric assessment (NGA), and **what** are their perspectives on decision-making about kidney replacement therapy while using this NGA? [94, 95]	Focus groups with patients, caregivers and healthcare professionals; purposive sampling; inductive approach using thematic analysis.
**What** are the perspectives of nephrologists regarding the ways in which family members facilitate or impede decisions to appropriately forego or withdraw dialysis treatment? [41]	Individual semi-structured interviews with healthcare professionals; purposive sampling; inductive approach using narrative analysis^b^ and thematic analysis.
**What** are experiences with and barriers for self-management from the perspectives of healthcare professionals and patients with limited health literacy, and **what** are strategies to optimize self-management? [64]	Individual semi-structured interviews and focus groups with patients and healthcare professionals; purposive sampling; inductive approach using thematic analysis and grounded theory analysis.
**What** are facilitators and barriers to translation of research into clinical practice for patients with autosomal dominant polycystic kidney disease? [71]	Individual semi-structured interviews with healthcare professionals; convenience sampling; inductive approach using thematic analysis.
**What** are the prognostic information preferences of older patients with chronic kidney disease? [62]	Individual semi-structured interviews with patients; purposive sampling; inductive approach using content analysis.
**What** are psychosocial barriers and facilitators for engaging in a healthy lifestyle among patients with chronic kidney disease, and **what** intervention strategies can be used to address these barriers? [33]	Focus groups with patients and healthcare professionals; purposive sampling; hybrid inductive approach (using thematic analysis) and deductive approach (using the existing theoretical domains framework).
**What** are the perspectives of transplant nephrologists and surgeons on the challenges and complexities of living kidney donor assessment? [44]	Individual semi-structured interviews with healthcare professionals; purposive sampling; inductive approach using thematic analysis and grounded theory analysis.
**What** are kidney transplant recipients’ needs and preferences for self-management support? [46]	Focus groups and individual semi-structured interviews with patients; purposive sampling; directed content analysis, using a deductive approach while also using inductive strategies (thematic analysis).
**What** are barriers and facilitators for discussing conservative management with older patients and nephrologists’ decisions to present the option of conservative management? [40]	Semi-structured interviews with healthcare professionals; purposive sampling followed by snowball sampling; inductive approach using narrative analysis^b^ and thematic analysis.
**What** are experiences of survivors of an acute kidney injury (AKI)-related hospitalization, **what** are barriers to shared and informed decision-making, and **what** are best practices for provision of patient-centered AKI care? [96]	Individual semi-structured interviews with patients; consecutive sampling; inductive approach using grounded theory analysis.
**What** are perspectives of patients and care partners on self-management in glomerular disease? [97]	Focus groups with patients and partners; purposive sampling; inductive approach using thematic analysis.
**What** are parents’ experiences of the genetic testing process, **what** is the psychosocial impact of genetic test results, and **what** are parents’ information needs and preferences regarding delivery of genomic information? [98]	Individual semi-structured interviews with parents of pediatric patients; purposive sampling; inductive approach using thematic analysis.
**How** satisfied are patients with their dialysis care, and **what** are their experiences and needs regarding advance care planning? [99]	Individual and duo semi-structured interviews with patients and family/friends; purposive sampling; inductive approach using grounded theory analysis.
**How** do patients and clinicians integrate principles of shared-decision making into discussions about vascular access, and **what** are barriers for predialysis vascular access care? [39, 70]	Individual semi-structured interviews with patients, caregivers, and healthcare professionals/personnel; purposive sampling; directed content analysis, using a deductive approach while also using inductive strategies (thematic analysis).
**How** do unspecified kidney donors experience the care within a transplant institute, and **how** can care be improved for this donor group? [100]	Individual semi-structured interviews with donors; consecutive sampling; inductive approach using thematic analysis.
**How** do nephrologists experience providing conservative care for patients with advanced chronic kidney disease? [101]	Individual semi-structured interviews with healthcare professionals; purposive sampling and snowball sampling; inductive approach using grounded theory analysis.
**How** do minority ethnic patients’ experience kidney end-of-life care, and **how** can services be delivered in a way that meets diverse patient needs? [65]	Focus groups and individual semi-structured interviews with patients and healthcare professionals; consecutive sampling; inductive approach using thematic analysis.
**How** do people on dialysis experience being on the waiting list for a kidney transplant from a deceased donor? [43]	Focus groups with patients; consecutive sampling; inductive approach using thematic analysis.
**Why** do some patients receiving hemodialysis treatment have difficulties with medication adherence and others do not? [45]	Individual semi-structured interviews with patients; purposive sampling; hybrid inductive approach (using thematic analysis) and deductive approach (using the existing World Health Organization determinants of medication adherence).
**Why** do patients with chronic kidney disease experience difficulties when trying to reduce their dietary sodium intake? [34]	Focus groups with patients and healthcare professionals; purposive sampling; hybrid inductive approach (using thematic analysis) and deductive approach (using the existing phases of behavior change of self-regulation theory).

a Based on existing qualitative research conducted within the field of nephrology; see references.

b Narrative analysis aims to understand how people create stories from their personal experiences.

Table 2 presents an overview of contrasting properties of quantitative and qualitative research. Please note that researchers can also adopt deductive (i.e. theory-driven/hypothesis-testing) and hybrid (i.e. inductive and deductive) qualitative approaches; for instance, to test whether themes derived from the data (i.e. induction) align with existing frameworks (i.e. deduction) [31–33]. To illustrate: to identify in which phases of behavior change patients with CKD needed self-management support, the ESMO study first identified perceived barriers for reducing sodium intake (i.e. induction) and thereafter organized them according to the existing behavior-change phases of self-regulation theory (i.e. deduction) [34].

**Table 2:** Contrasting properties of quantitative research and qualitative research.^a^

Property	Quantitative research	Qualitative research
Research aim	To test hypotheses and establish universal theories (e.g. about cause and effect and predicting the future).	To gain an in-depth understanding of complex social phenomena in the context in which it occurs.
Research question	Research questions to confirm and quantify, starting with: Whether? How much? How often? How strong? Etc.	Research questions to find out what is going on (explore and explain), starting with: What? How? Why? Etc.
Type of reasoning	Deductive reasoning (i.e. theory/hypothesis-testing): to test existing theories through hypothesis-based research questions comprising predetermined variables.	Inductive reasoning (i.e. theory-building/hypothesis-generating): to develop rich descriptions, conceptual frameworks, and/or explanatory theories directly from the data.
Data type	Numeric data.	Non-numeric or naturalistic data.
Data collection	Predetermined data using standardized processes and instruments (e.g. data from electronic health records and answers to closed-ended survey questions).	Written answers to open-ended survey questions, audio (or video) recordings from interviews, transcripts, field notes, objects, images, documents, etc.
Sampling	Relatively large (random) samples, selected using probability sampling methods, in order to minimize bias and maximize statistical representativeness of populations.	Relatively small samples, selected using non-probability sampling methods, in particular purposive sampling to ensure a nuanced and comprehensive understanding of topics.
Perspective on topic(s) at hand	Reductionistic and relatively simplified perspective on the topic(s), and the topic(s) are explored from the researcher's perspective.	Holistic, open and in-depth perspective on the topic(s), and the topic(s) are explored from the participant's perspective.

a For clarification purposes, all properties of quantitative and qualitative research have now been presented as contradictions. In practice, these properties are not looked upon as being so binary but rather as a spectrum (e.g. some qualitative methods may also include deductive approaches while some quantitative methods also employ inductive approaches; see also **What**).

Qualitative research can be conducted as stand-alone research but can also be combined with quantitative research in a ‘mixed-methods’ design. Qualitative and quantitative methods answer different research questions and thus combining one or multiple qualitative and quantitative methods makes it possible to complement each other's limitations and capitalize on each other's strengths (see also **Why**). For example, qualitative methods are used to generate new hypotheses, while quantitative methods are used to confirm these hypotheses and build on this evidence [9, 26, 35, 36]. To illustrate: the ESMO study built on the aforementioned qualitative evidence by conducting a survey in a larger sample to investigate which sodium barriers were important and which factors were associated with experiencing sodium barriers [37]. All results were hereafter incorporated into the ESMO trial design, a trial showing that a self-management intervention—addressing the most important barriers and associated factors—can improve health outcomes [i.e. sodium excretion, blood pressure, protein excretion and self-efficacy (an individual's belief in their capacity to manage the disease)] of patients with CKD [21].

## WHY: WHY CAN QUALITATIVE RESEARCH BE OF VALUE IN THE CLINICAL AND EPIDEMIOLOGICAL (NEPHROLOGY) CONTEXT?

While clinical and epidemiological research significantly enhances evidence-based healthcare, it often leaves important gaps in our understanding of health and illness. Qualitative research has been recognized as a key to addressing some of these gaps, using both exploratory (to gain a more complete and in-depth understanding of problems) and explanatory (to explain quantitative results) approaches [6–9, 12, 13, 26, 36]. Below are listed various reasons why qualitative research can be of value, including different qualities that can also strengthen each other (see also Table 3).

**Table 3:** Overview of the value of qualitative research in the clinical and epidemiological context.^a^

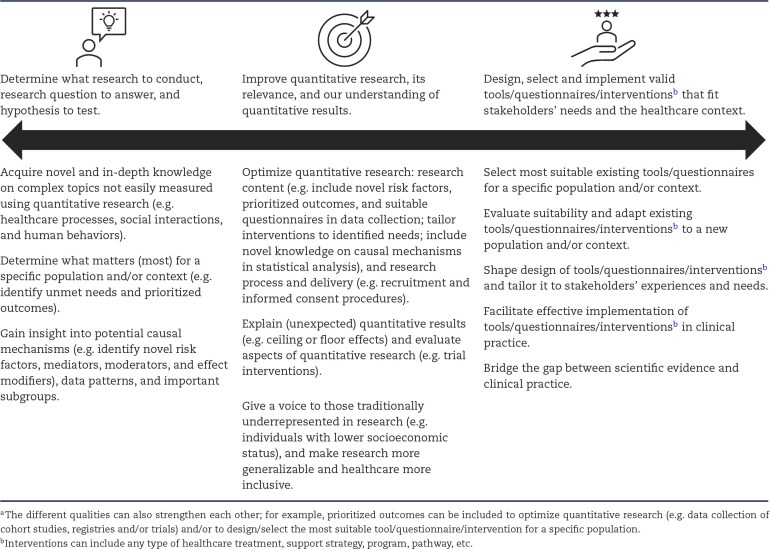

### A. Determine what research to conduct, which questions to answer, and which hypothesis to test

Qualitative research can step beyond what is already known and explore the world from a diverse non-researcher perspective. In doing so, it makes discoveries (e.g. novel insights, prioritized outcomes and causal mechanisms) that can inform quantitative research, with examples presented below [4, 5, 26, 27, 38].

#### Acquire novel and in-depth knowledge on complex topics

Novel in-depth knowledge can be obtained on complex topics not easily measured by quantitative methods, like healthcare processes, social interactions and human behaviors [17, 26]. Within such topics, the focus often lies on identification of patients’, caregivers’ and healthcare professionals’ experiences, perspectives, feelings, expectations, preferences and needs, as they drive and explain people's responses to illness, behaviors, treatment effects and health outcomes [4–9]. For instance:

- Medical decision-making: experiences with integrating shared-decision making into discussions about vascular access [39]; experiences, beliefs and challenges when discussing conservative management with older CKD patients [40]; and perspectives on family involvement in decisions to forego or withdraw dialysis [41].
- Kidney donation: expectations, attitudes and beliefs of older recipients living with a kidney transplant [42]; experiences with living on dialysis while waiting for a kidney transplant [43]; and challenges, uncertainties and controversies regarding living kidney donor assessment [44].
- Adherence: impeding and facilitating factors for medication adherence [45]; and self-management challenges and support needs among kidney transplant recipients [46].

Such knowledge can inform future research, clinical care, and the development of successful healthcare tools, questionnaires and interventions (see also **B/C**) [7, 20, 47].

#### Determine what matters (most) for particular populations and contexts

By offering insight into stakeholder stories, it is possible to identify needs and priorities; for instance, identify unmet needs of older patients receiving hemodialysis [48], and transportation burden and needs associated with hemodialysis [49]. Priorities can also be elicited for healthcare developments and research, e.g. patients’ priorities regarding remote management technologies for peritoneal dialysis [50], CKD research topics [51] and health outcomes for nephrology trials [52]. Such knowledge can inform future research (e.g. address unmet needs with interventions and evaluate its effectiveness using prioritized outcomes; see also **B/C**) and how to care for patients [18].

#### Gain insight into potential causal mechanisms, patterns and subgroups

Qualitative research can improve and broaden our clinical and epidemiological insights [53, 54]. First, it can reveal potential causal mechanisms underlying outcomes and generate hypotheses about such mechanisms. Potential new causes can be identified as qualitative research looks beyond what is already known (e.g. participants may describe determinants not yet found in literature) and takes into account social factors and contexts (e.g. thereby identifying psycho-socio-cultural determinants) [53, 54]. It can also help researchers to understand, from participants’ perspectives, how associations work (i.e. mediators), what affects the strength and/or direction of associations (i.e. moderators), and when associations differ in circumstances (i.e. effect modifiers) [53, 54]. For example, recent qualitative findings suggest that optimism may play a moderating role in the association between patients’ perceptions about their CKD (i.e. illness perceptions) and health outcomes [32]. Mapping options from qualitative analysis software can even assist in visualizing hypothesized chains of causal effects by creating directed acyclic graphs (DAGs) [54]. Second, qualitative research can identify patterns (e.g. events happening in a certain order) and help to understand how these patterns may influence outcomes. This is especially relevant in healthcare research, as such outcomes and events can rarely be explained without taking into account perceptions, attitudes and behaviors of the individuals involved [20]. Furthermore, population-level data may obscure subgroup differences and their meaning within a population. Qualitative research can identify important subgroups, thereby informing future research and statistical analysis [38]. To illustrate the above two points, findings of the aforementioned qualitative research also suggest that two critical moments precede changes in illness perceptions (namely, receiving the CKD diagnosis and receiving the message that kidney replacement therapy needs to be initiated soon) and that younger patients more often had unhelpful negative mindsets [32].

### B. Improve quantitative research, its relevance, and our understanding of quantitative results

Qualitative research can contribute to improved quantitative research, its relevance, and our understanding of (unexpected) quantitative results, with examples presented below.

#### Optimize design of quantitative research

Qualitative evidence can contribute to research design optimization. Quantitative research can be improved by incorporating qualitative evidence (see also **A/C)** into their design; for example, observational (cohort) studies can add novel identified risk factors to their data collection; clinical trials can tailor interventions to identified needs of patients, evaluate its effectiveness in terms of outcomes prioritized by patients, and use suitable questionnaires for the respective population and context; and statistical analysis can include novel knowledge on causal mechanisms and important subgroups [20, 54, 55]. Research process and delivery can also be improved, e.g. pre-trial interviews leading to improved trial recruitment (e.g. leaflets), consent procedures and presentation (e.g. trial name and arms presentation), examples can also be found in nephrology [56, 57]. In doing so, relevance and accuracy of quantitative results can be improved and design issues can be addressed in advance [20, 54].

#### Explain quantitative results and evaluate quantitative research aspects

Qualitative research can explain (unexpected) quantitative results. For instance, it can explain and contextualize findings from surveys and statistical modeling (e.g. ceiling or floor effects) and from trials (e.g. why and how interventions had an impact or not) [20, 26, 38, 58]. It can also be used to evaluate certain quantitative research aspects, such as tools and questionnaires in implementation research, e.g. to evaluate how patients experience the use of PROMs as part of routine dialysis care [20, 59]. Moreover, post-trial interviews are often used to evaluate trial participants’ experiences with (effects of) interventions and their beliefs about personal benefits and harms [20, 60]; for example, in patients with autosomal dominant polycystic kidney disease, their overall experiences, expectations, motivations for enrollment, staff interactions, and recommendations for future trial design [61]. Such knowledge can improve future trial research and facilitate effective implementation in practice (see also **C**) [60].

#### Give a voice to those traditionally underrepresented

Qualitative research enables inclusion of populations traditionally underrepresented in research, such as individuals who are older, with lower socioeconomic status or literacy levels, and from minority ethnic backgrounds. For them, qualitative methods (e.g. interviews) may be more suitable, effective and less intimidating than other methods (e.g. surveys) [26]. Various examples exist: prognostic information preferences of older CKD patients [62]; needs concerning home-based support among low socioeconomic status older adults treated with hemodialysis [63]; perceived self-management barriers of CKD patients with low levels of health literacy [64]; and minority ethnic patients’ experiences and needs regarding kidney end-of-life care [65]. By including those who have been historically marginalized, important knowledge is obtained (e.g. prioritized outcomes and unmet needs in these populations), and important steps can be made towards making research more generalizable and healthcare more inclusive.

### C. Design, select and implement valid tools, questionnaires and interventions that fit stakeholders’ needs and the healthcare context

Qualitative research can contribute to using suitable patient-centered approaches to measure and improve health outcomes in routine clinical care, with examples presented below [7, 20, 26].

#### Select the most suitable existing tools and questionnaires

Qualitative research can be used to select the most suitable existing tools or questionnaires (e.g. to measure patient-reported outcomes) for particular populations or contexts, e.g. to select the most valid PROM for routine symptom assessment in patients with advanced CKD, which has been successfully implemented into Dutch dialysis care [59, 66].

#### Evaluate suitability and adapt existing tools, questionnaires and interventions

This can be applied to evaluate suitability for and adapt existing tools, questionnaires and interventions to new populations and/or contexts, for instance using cognitive interviewing and think-aloud sessions to capture participants’ thought processes while engaging in activities (e.g. reading patient education or filling in questionnaires). For example, the suitability and content validity of Illness Perception Questionnaires have been evaluated, resulting in various suggestions to adapt these questionnaires to patients’ experiences with CKD [32, 67].

#### Shape design of tools, questionnaires and interventions

An in-depth understanding of people's needs and lived illness experiences within their social and healthcare contexts (and the meaning they attribute to these experiences) is a crucial determinant for designing tools, questionnaires and interventions and their success [20]. This holds especially true for patients with chronic (kidney) conditions and various examples exist of pre-trial interviews informing treatment design. For instance, identify support needs, barriers and facilitators for adherence to a healthy lifestyle [33, 34]—qualitative evidence that has been successfully incorporated into CKD self-management interventions of the E-GOAL, SUBLIME and ESMO trials [21, 68, 69].

#### Facilitate implementation of tools, questionnaires and interventions in clinical practice

Qualitative research is essential to facilitate effective implementation of tools, questionnaires and interventions in clinical practice [7]. To this end, feasibility, acceptability and appropriateness should be explored prior to implementation and potential barriers for successful implementation can be identified. Various nephrology examples exist, e.g. exploring feasibility and acceptability of a home-based program to improve functioning of low socioeconomic status older adults treated with hemodialysis [63] and identifying potential barriers for effective implementation of a vascular access support program [70], with both studies adjusting their protocols accordingly thereafter.

#### Bridge the gap between scientific evidence and clinical practice

Qualitative research can help to bridge the gap between scientific evidence and clinical practice [7]. Several studies have focused on gathering in-depth knowledge on how to translate scientific evidence into practice, e.g. facilitators and barriers for translating clinical research results into routine care for patients with autosomal dominant polycystic kidney disease [71]. Moreover, qualitative research should not only be regarded as hypothesis-generating; some results can be directly used in clinical practice [31]. For example, qualitative evidence on patients’ and healthcare professionals’ experiences and perspectives with regard to collecting PROMs and discussing PROMs-results led directly to practical recommendations for routine dialysis care [28, 59].

## HOW: HOW TO DEVELOP QUALITATIVE RESEARCH IN THE CLINICAL AND EPIDEMIOLOGICAL CONTEXT

Similar to quantitative research, qualitative research should be well designed and have a clear and reasonable rationale (see also **Why**), and key methods elements should be predetermined. Qualitative research consists of a broad collection of approaches—too many to discuss in one article. Therefore, we present an overview comprising basic steps and considerations of the most commonly used and applicable strategies within clinical and epidemiological (nephrology) contexts (e.g. inductive approaches and open-ended interviews) [7, 8, 18, 26, 31, 72, 73]—important information that should also be reported in qualitative research protocols (see Table 4 for a self-developed checklist and Table 1 for examples of basic methods elements used in nephrology).

**Table 4:** Self-developed checklist for important information to be included in qualitative research protocols.^a^

**The following information has been included** **^b^** **in this qualitative research protocol:**
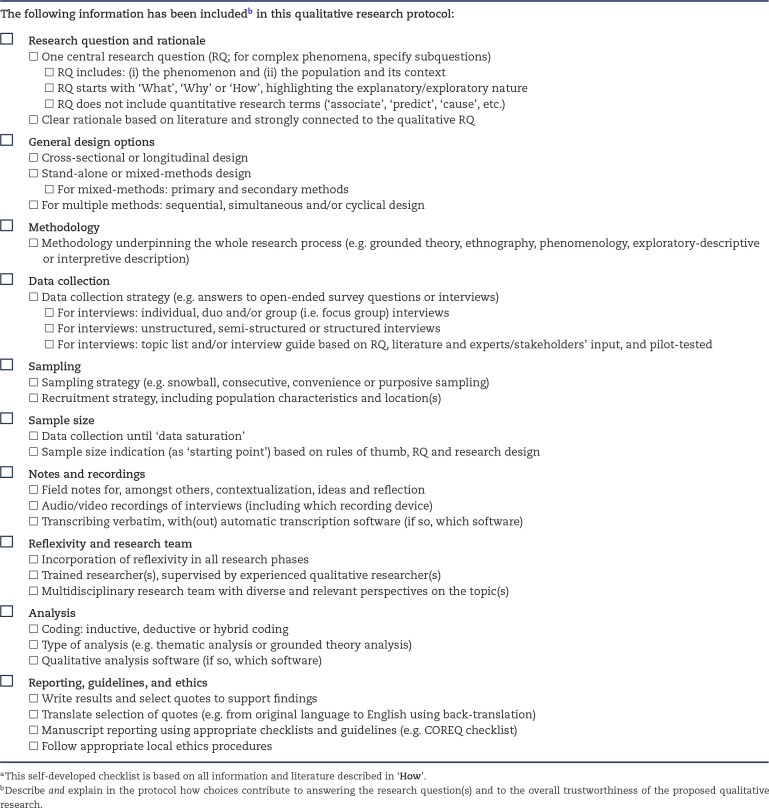

### Research questions and rationale

As most qualitative research aims to get an ‘in-depth understanding of a social phenomenon in the context that it occurs’ [16], research questions should at least contain: (i) the phenomenon it aims to understand, and (ii) in which population and context. Usually, there is one central question, but for complex phenomena subquestions can be specified. Following its commonly exploratory and explanatory nature, questions usually start with ‘What’, ‘Why’ and ‘How’, and do not contain quantitative research terms (‘associate’, ‘predict’, ‘cause’ etc.; see also **What/**Table 1) [74]. Finally, a strong rationale is fundamental and should be clearly formulated, based on and put in context using existing scientific literature, and should be directly connected to the research question (see also **Why**/Table 3).

### General design options

Depending on the research question, different general design options are available. First, whether to conduct stand-alone qualitative research and, if so, to use a cross-sectional (i.e. data collection at a single point in time) or longitudinal (i.e. repeated data collection, e.g. exploring changes over time) design. For longitudinal design instructions, see Saldaña [75]. When combining qualitative research with quantitative research in a mixed-methods design (see **What**), the weight given to each method can differ: one may use both methods equally, or give more weight to one method (i.e. primary method) than the other (i.e. secondary method). Furthermore, when using multiple (qualitative and/or quantitative) methods, they can be employed sequentially (i.e. one method used after the other), simultaneously (i.e. two methods used at the same time) and/or cyclically (i.e. one method repeated after completion of the other) [9]. For more details on mixed-methods design options, see Bailey *et al*. [36].

### Methodology

‘Methodology’ refers to the overarching approach (i.e. tradition) underpinning the whole research process. Not to be confused with ‘methods’, which refers to the actual data collection and analysis strategies. Common methodologies, which share similarities, but have different aims, include grounded theory (inductive development of theories about social phenomena through systematic, iterative data collection and analysis), ethnography [description and interpretation of (behaviors of) social groups and cultures], and phenomenology (exploration and description of how people make sense of the world and their lived experiences). Other common methodologies in healthcare include exploratory-descriptive and interpretive description, the latter seeking to capture experiences and perspectives from clinical practice to generate in-depth knowledge to enhance clinical practice [31, 76, 77]. For more details on methodologies, see previous publications [8, 78].

### Data collection

Different data collection strategies exist, including observations, collection of images, documents and responses to open-ended survey questions. The most common strategy to collect data rich in details and context is open-ended discussions: individual interviews (e.g. with a patient), duo interviews (e.g. with a patient and partner) and focus groups (i.e. interviews with a small group of participants, e.g. healthcare professionals) [8, 73]. Each interview has its purpose and approach, and thus results in different data:

- Individual interviews allow more focused discussions, exploring different facets of topic(s) using follow-up questions and without interruptions. This provides opportunities to unravel participants’ thinking and reasoning within their context and results in deeper insight into participants’ perspectives. It is also suitable to explore personal, sensitive or controversial topics that participants feel less comfortable discussing in groups [31, 74].
- Focus groups (and to a lesser extent also duo interviews) are all about ‘interaction’: one group participant's perspective feeds off another participants’ perspectives (by listening to it, thinking about it, responding to it, questioning or challenging it, adding to it, (dis)agreeing with it, etc.); discussions continue to evolve through interaction and reflection, and thus result in data created by (group) interaction. It should be used when this (group) dynamic has added value—to explore broad topics from multiple perspectives, ‘how people talk’ about certain topics, (the lack of) consensus, etc. Moreover, it is ideal for exchanging opinions and viewpoints, and for brainstorming activities leading to new ideas and solutions to particular problems [31, 74].

Depending on the research question, one strategy or multiple strategies can be used.

Different interview structures exist, ranging from unstructured (i.e. starting with only general topics related to the research question) to structured (i.e. starting with a predetermined list of open-ended questions with no adaptions possible). Semi-structured is most often used: starting with a list of (sub)topics and open-ended guiding questions (i.e. ‘interview guide’) that should be covered to answer the research question. The interview schedule and process are flexible in terms of number and order of questions, language, use of follow-up/clarifying questions, etc. Topic lists and interview guides should be pilot-tested and based on the literature, and supplemented with experts’ and stakeholders’ input [20, 31].

### Sampling

Different types of non-probability sampling can be used, including snowball (i.e. participants recruit future participants from their network), consecutive (i.e. select participants who meet the inclusion criteria and are conveniently available), and convenience (i.e. select participants solely because they are conveniently available) sampling. Purposive sampling is most widely used, allowing researchers to select participants with specific characteristics (e.g. reflecting the population's diversity) and information-rich participants (i.e. offering valuable perspectives on the research question), thereby ensuring a nuanced and comprehensive understanding [72, 73]. Recruitment can take place at a single location or multiple location(s) to increase the results’ transferability (i.e. its external relevance). For details on sampling and recruitment, see Lopez and Whitehead [79] and Negrin *et al*. [80].

### Sample size

Qualitative research is an iterative process, meaning that researchers do not follow a linear path but continuously cycle back and forth between research stages. Also between data collection and analysis: preliminary results inform researchers to continue data collection until ‘data saturation’ is reached—the point at which additional recruitment yields no or minimal new information for the research question. Thus, no a priori sample sizes exist for qualitative research because the sample size depends on the nature (i.e. ‘richness’) of the data [18, 81, 82]. Commonly used rules of thumb do exist; these rules suggest starting with 20 individual interviews (often lasting about 1 hour) and three or four focus groups (with approximately 6–12 participants, often lasting about 2 hours) [26, 31, 72, 73, 83]. Please note that to compare subgroups (e.g. younger versus older participants), a substantially larger (e.g. double) sample is needed to reach saturation within and between groups [84].

### Notes and recordings

Field notes are written to contextualize data collection and reflect on the entire research process: observations about interviews, general impressions of participants, personal reflections [on personal biases (see **Reflexivity and research team**)], thoughts about identified data patterns, etc. For guidance on field notes, see Phillippi and Lauderdale [85]. With participant consent, interviews are audiotaped (or videotaped) to ensure accurate data collection and enable researchers to focus on the discussion. All recordings are transcribed verbatim, meaning transcripts are exact reproductions of interviews (without privacy-sensitive information). Software exists to support transcribing (e.g. Amberscript), but make sure to only use software guaranteeing privacy and to check data for errors.

### Reflexivity and research team

Qualitative research is inherently subjective and the researcher's subjective perspective is fundamentally intertwined with the whole research process. Acknowledgment that qualitative research depends on subjectivity is an important step in becoming a qualitative researcher. To account for how subjectivity shapes the research, it is vital to incorporate reflexivity into all research phases. Reflexivity is the process of active engagement in continuous critical examination and reporting about oneself as a researcher (e.g. your conceptual lens, background, biases, preferences, preconceptions, values, and context) and the research relationship (e.g. with participants), and how your subjectivity and biases guide and inform the research process and could impact results [86]. For a practical guide to reflexivity, see Olmos-Vega *et al*. [86]. Given that the research quality is so heavily dependent on the individual researcher (skills, personal biases, idiosyncrasies, etc.), it is essential that the research is executed by researcher(s) trained in qualitative research, under the leadership of an experienced qualitative researcher and in close collaboration with a multidisciplinary team—a triangulation strategy (i.e. input of multiple methods and sources) to ensure results are trustworthy [6, 72]. Team discussions are especially important to ensure consistency in analysis strategies and to reach consensus on interpretations of qualitative data.

### Analysis

The data analysis strategy depends on the methodology and research question [72]. Many approaches follow the broad basic precepts of inductive coding and thematic (content) analysis [8, 87, 88]. This means that, after familiarization with and close reading of transcripts, an iterative bottom-up approach is taken: (i) initial codes are generated inductively: all meaningful text fragments are identified and assigned a code (i.e. a label of one or multiple words) capturing its meaning in relation to the research question; and (ii) similar codes are grouped into (sub)themes based on (patterns of) shared meaning concerning the research question [17]. Practical guides are available, for instance for thematic analysis (Braun and Clarke [88]) and grounded theory analysis (Birks and Mills [89]). Finally, software (e.g. Atlas.ti and Nvivo) can facilitate proper data management, auditable and efficient data analysis, and visualization of results (e.g. code trees, thematic maps). Qualitative analysis is often considered labor-intensive and time-consuming, requiring constant movement between immersion, examination and interpretation of a great volume of data (e.g. interview transcripts), coding, creation of themes, team discussions, etc.

### Reporting, guidelines and ethics

Results are written and illustrative quotes are selected to support the main findings. If necessary, quotes are translated, for example using back-translation: text in the original language is translated to English by one researcher and translated back (i.e. from English to original language) by a second researcher to ensure accuracy. Guidelines exist to ensure complete and transparent reporting in manuscripts, including the Consolidated criteria for Reporting Qualitative research (COREQ) checklist [90]. Appraisal guidelines for manuscripts also exist, providing information on the four criteria for establishing overall trustworthiness and strategies to ensure scientific rigor and mitigate bias: (i) credibility: does it offer comprehensive, trustworthy, and sensible explanations for findings? (ii) dependability: is it conducted in a rigorous and systematic manner that is auditable? (iii) transferability: are findings relevant to other contexts? and (iv) confirmability: do the findings reflect participants’ perspectives without being influenced by researchers’ biases, assumptions, agendas, etc.? [72, 91, 92]. Finally, follow all appropriate local ethics procedures for conducting qualitative research.

## CONCLUSIONS

We have aimed to equip clinicians, clinical researchers and epidemiologists with the basic knowledge to appraise and design qualitative research. A summary of key aspects has been given in the different tables: qualitative research questions with commonly used methods elements (Table 1), the properties of qualitative research (Table 2), the value of qualitative research in the clinical and epidemiological context (Table 3), and basic steps and considerations for writing qualitative research protocols (Table 4). We encourage clinical researchers and epidemiologists to apply the qualitative methods discussed in this article to enrich their research with complementary qualitative evidence and to contribute to evidence-based healthcare (e.g. by providing systematic and high-quality substance to the pillar ‘patient values’ and the pillar ‘clinical expertise’). Future research should especially focus on developing mixed-methods research that leverages the strengths of both qualitative and quantitative research to address complex healthcare problems.

## Data Availability

No new data were created or analyzed. Data sharing is not applicable to this article.
